# Characterizing the Dynamic Textural Properties of Hydrocolloids in Pureed Foods—A Comparison Between TDS and TCATA

**DOI:** 10.3390/foods8060184

**Published:** 2019-05-30

**Authors:** Madhu Sharma, Lisa Duizer

**Affiliations:** Department of Food Science, University of Guelph, Guelph, ON N1G 2W1, Canada; madhu@uoguelph.ca

**Keywords:** temporal dominance of sensations (TDS), temporal check-all-that-apply (TCATA), pureed foods, carrots, starch, xanthan, oral processing, dynamic perception, International Dysphagia Diet Standardization Initiative (IDDSI)

## Abstract

Pureed foods, a compensatory diet for dysphagia, require the incorporation of hydrocolloids in order to be swallowed safely. The effect of hydrocolloid addition on textural dynamics of pureed foods has not yet been investigated. Starch and xanthan were added to levels that allowed products to meet the criteria of the International Dysphagia Diet Standardization Initiative. Nine pureed carrot matrices made with two concentrations of starch, xanthan, and their blends were characterized for textural evolution using two dynamic sensory techniques: Temporal Dominance of Sensations (TDS) and Temporal Check-All-That-Apply (TCATA). Each test, with four replications, was conducted with 16 panelists. Results indicate that purees were divided into two groups based on sensory responses––grainy and smooth were the primary differentiating attributes for these two groups. Grainy was associated with starch-added samples, while samples with xanthan (alone and in blends) were smooth and slippery. For both groups, thickness was perceived during the first half of processing, adhesiveness in the second half of oral processing, and mouthcoating was perceived toward the end of processing. A comparison of results from these tests showed that both TDS and TCATA gave similar information about texture dynamics and product differentiation of pureed foods.

## 1. Introduction

Texture modified foods, such as pureed foods, are a recommended management option for people with swallowing disorders, particularly those with oropharyngeal dysphagia [1]. Pureed foods are semisolid materials wherein the food structure has been destroyed during blending and pureeing. Liquids can be added to ensure proper consistency [2]. Hydrocolloids are also added and form an integral part of these foods. Hydrocolloids improve product consistency and cohesiveness and reduce syneresis of the product [3,4,5]. These improvements make the food safe to swallow for individuals with dysphagia. While providing the desired benefits, hydrocolloids will affect the rheological and sensory properties of the food [6,7]. They impact food microstructure, the breakdown of particles, force needed to deform the food during mastication, lubrication of the bolus, and mouthcoating [8,9]. Each of these properties impact food oral processing and ultimately sensory perception. Therefore, the effect of hydrocolloid addition on sensory perception of foods must be measured.

The two most commonly used hydrocolloids added to dysphagia-specific products are starch and xanthan [10,11]. At present, sensory studies examining the addition of these hydrocolloids to products for individuals with dysphagia have primarily focused on thickened fluids [12,13,14,15,16]. When starch or xanthan are added to liquids, they interact with salivary components, resulting in differences in sensory perceptions [3]. Starch imparts a grainy texture in thickened liquids while xanthan-thickened liquids are perceived as sticky and slippery [12,14]. There is less known about the effect of hydrocolloid addition on perception of semi-solid foods, particularly pureed foods. In one of the few published studies examining modified textured foods, xanthan-thickened pureed carrots were perceived as smooth, sticky, and slimy while starch thickened carrots were rated low in smoothness [17]. Starch addition also produced a product that was perceived to be denser than the xanthan-thickened product [18]. However, all of these sensory results are static and the dynamic perceptions of these foods have not yet been characterized. As food structure and associated properties change during oral processing, their perceptions also evolve and keep changing as the food is mixed with saliva, manipulated, and prepared to form a swallow-able bolus.

A number of sensory methods exist for measuring dynamic nature of perception. Two recent techniques are Temporal Dominance of Sensations (TDS) and Temporal Check-All-That-Apply (TCATA). TDS and TCATA differ in their approach. TDS is based on the philosophy of capturing the most dominant sensation at a particular time whereas TCATA captures all the sensations being perceived at a particular time, during oral processing. The advantages of TDS and TCATA over other dynamic sensory tests are that both are less time-consuming, require minimal training of judges, and can be used for up to 10 attributes during each evaluation [19,20,21]. The methodology and analysis of TDS data was first introduced in 2003 and since then it has been extensively used in almost all oral processing studies to understand the dynamics of sensory perception [22]. This has included examining flavors, tastes, and textures of semi-solid gel-based systems made with different sweeteners [23,24]. Textures of emulsion-filled gels have also been studied using this approach [25]. Using the technique of TDS, Varela et al, (2014) examined how hydrocolloid addition modulates the negative attributes in ice cream, e.g., iciness [26]. TCATA is a relatively recent technique, introduced in 2016 and has been used to examine flavors and taste in aqueous model systems with non-nutritive sweeteners [27,28]. While these two techniques provide a holistic picture of the dynamics of sensory attributes of a food as they evolve and change during oral processing, few studies have compared the results of these two techniques [29,30,31]. Those that have been conducted have not examined semi-solid foods.

Texture, the primary attribute responsible for flow of the bolus, is the driving factor for safety during swallowing of modified textured foods. The purpose of the current research was two-fold. The first objective was to investigate the effect of starch, xanthan, and their blends on dynamic texture perception in a hydrocolloid thickened pureed food. It is hypothesized that since the original texture of the food is destroyed during pureeing, the type and concentration of added hydrocolloid contributes significantly to textural properties. The second objective was to determine if TDS and TCATA provide similar or complementary information in terms of texture evolution and product differentiation considering the aspect of dynamics of sensory perception.

## 2. Materials and Methods

In this study, dynamic testing of semisolid foods was conducted using pureed food matrices made with two hydrocolloids and their blends. Pureed carrots made with starch and xanthan were studied for temporal evolution of texture attributes. Carrots, being part of orange colored vegetables, were selected as a model food because they are recommended for older adults (with or without swallowing difficulties) as a good source of fiber and Vitamin A [32,33]. The carrots (cultivar SV2384DL, Stokes Seeds, Canada) were generously donated by the Muck Crops Research Station, Guelph, Canada. The starch, Precisa Sperse 100, was provided by Ingredion Inc. (Bridgewater, NJ, USA). This is a cold water swelling modified waxy maize starch, indicative of a high amylopectin content. Xanthan, KELTROL®-521, was supplied by CP Kelco (Atlanta, GA, USA). It is an 80-mesh xanthan gum with low dusting characteristics and a high hydration efficiency. All carrots were cooked, pureed, and vacuum sealed in plastic bags and stored in a freezer (−20 °C) until required. To prepare the puree, carrots were peeled, sliced, and pressure cooked in an electric pressure cooker (Power Pressure Cooker XL, Tristar Products Inc., Model-PPC772) for 4 min (under the settings ‘vegetables’), at a pressure of 70 kPa. For cooking, 80 g of water was added to 1 kg of peeled, sliced carrots. After cooking, carrots were strained and cooled for 15 min and pureed in a Robot Coupe food processor (R2NCLR, 1725 rpm) for 15 min with intermittent breaks, every 3 min, for stirring and mixing. The pureed carrots were vacuum sealed using a FoodSaver® 2-in-1 Vacuum Sealer and immediately frozen (−20 °C) until further use.

The pureed carrot matrices with added hydrocolloids were prepared on each day of sensory testing. The frozen pureed carrots were defrosted for 14 hours in a refrigerator (4 °C). Prior to mixing the hydrocolloids, the temperature of the purees was equilibrated to room temperature (approximately 20 °C). Starch/xanthan were added to the puree and mixed using a whisk attachment in KitchenAid Artisan Mixer (325 W) for 80 s with intermittent breaks, after every 20 s, for scraping the puree from sides in order to have a uniform dispersion of hydrocolloid in the carrot puree. The control sample with no additives was also subjected to similar processing, in order to maintain consistency. The product was then transferred to Styrofoam cups and warmed to 55 °C in a temperature-controlled cabinet (Metro C5 Controlled Temperature) before being served for the sensory testing.

The concentrations of starch (0.8% *w*/*w*) and xanthan (0.4% *w*/*w*) added to the purees were selected such that the purees would meet International Dysphagia Diet Standardization Initiative (IDDSI) guidelines for modified textured foods. A broad outline of the four tests which were followed as part of IDDSI guidelines has been included in the Supplementary Section. Pureed foods fall under IDDSI level 4 (Table S1). Using the selected concentrations as the maximum level, a 3 by 2 full factorial design with 9 treatments (Table 1) was selected to investigate the effect of type and concentration of each hydrocolloid and their blends, on the dynamic sensory texture properties of pureed carrots.

### 2.1. Attribute Selection and Training

Two dynamic sensory tests were conducted on the puree treatments: Temporal Dominance of Sensations (TDS) and Temporal Check-All-That-Apply (TCATA). Both tests were conducted with same attributes. The attributes selected for dynamic texture evaluation are usually based on previous sensory studies of similar products [30,34]. A list of nine attributes was generated for this study based on the results of projective mapping and a trained panel examining textural perceptions in pureed carrot matrices [17,18]. Attributes selected include: adhesive, cohesive, dense, grainy, mouthcoating, slippery, smooth, thick, and thin.

The TDS and TCATA tests were completed by 16 panelists each and four replications of each test were conducted. All panelists (age between 20–40 years) were recruited from the University of Guelph. Selection was based on the availability and interest of panelist in the study. The TDS panel consisted of two males and 14 females and the TCATA panel consisted of three males and 13 females. Two panelists completed both tests. Tests were conducted 5 months apart.

This study was approved by the University of Guelph Research Ethics board (REB # 17-04-013). All subjects gave their informed consent for inclusion before they participated in the study. For each test, panelists were oriented for 2 days, 1 h each day. The purpose of this orientation was to familiarize all panelists with the selected attributes, the concept of dynamic testing, and the use of computer software for sample evaluation. On the first day, each attribute was defined and explained using one reference food (Table 2). This was done so that the panel developed a common understanding of attributes found in a pureed carrot matrix. It was clearly stated to the panel that there were no restrictions on the selection of the number of attributes and also the fact that the same attributes could be re-selected if perceived again. The concept of the test—TDS (the sensation/attribute that triggers the attention most) and TCATA (check/uncheck all the attributes being perceived or stopped being perceived) was exemplified by explaining how the attributes change over time while eating a hard cookie—starting from hard and crunchy, then moving to soft and sometimes pasty towards the end.

Oral processing of a pureed food is short; therefore, the layout of attribute list was kept the same for all training and testing days so that the panelists were aware of attribute location on the screen. The attribute layout was similar for all the panelists and it was same for both TDS and TCATA. Testing was conducted on individual laptops and the data was collected using Compusense Cloud version 8.8 (Compusense Inc., Guelph, ON, Canada). The panelists were instructed to eat the whole sample (10 ± 1 g) served in the Styrofoam cups for sensory evaluation. The cups were coded with three-digit blinding codes. They were required to cleanse their palate with a bite of unsalted cracker and a sip of water after each sample. The samples were randomized across all panelists and across all replicates for each panelist.

### 2.2. Data Analysis

The data collection and analysis were similar for both the tests. First, TDS and TCATA product curves were constructed by plotting dominance (TDS)/absence or presence (TCATA) of each attribute by standardized time. For TDS curves, two additional lines were drawn for statistical interpretation. The first line, ‘chance level’ represents the selection of an attribute by random, was set at 0.11 (1/9). The second line ‘significance level’ represents statistical significance (95% confidence) for an attribute and was calculated as 0.18 [35]. An attribute is said to be significantly dominant at a given point in the mastication process if its proportion of selection is above this line of significance for that time period.

To examine the hypothesis that type and concentration of thickener will have an effect on dynamics of perception, difference curves, based on Fischer’s Exact test (95% significance), were generated using Compusense. This was done on the non-standardized data, representing the actual oral processing time (OPT).

A product map was generated by conducting Canonical Variate Analysis (CVA), using the CVApack R-package [36]. In this two-way multivariate analysis of variance, samples were taken as fixed effect and panelists as random effect. The outcome is a product map with confidence ellipses for each sample, representing subject ellipses, where there is a 90% probability of specific sample selection by a subject. The layout of the ellipses and segment lines connecting samples give information about the degree of sample similarities and differentiation. The information on this map is a statistical test that complements the results shown on the product curves. To investigate the effect of hydrocolloid type and concentration on chewing time, oral processing times were determined and an Analysis of Variance (ANOVA—repeated measures) was conducted with samples as a fixed factor and panelists as a random factor. A Tukey’s Honest Significant Difference (HSD) with a level of significance of *p* < 0.05 was performed to check the samples which differed significantly from one another.

A trajectory map, representing the oral breakdown path of each sample, was constructed by conducting a Principal Component Analysis (PCA) on the TDS and TCATA standardized data [37]. It gives information about progression of perceived attributes from the initial point of ingestion of food until it is swallowed. This was analyzed with the tempR package in R software version 3.5.1 using a data table where rows represented the dominance rate/citation proportion of each sample at 11 time-points, starting from 5%, 10%, 20%,…, to 100% and the attributes tested in columns [38]. In this research, there were 9 columns (attributes) and 99 rows (11 time-points × 9 samples). The sensory trajectory was drawn by linking the 11 points of a particular sample from 5% (initial chew time) to 100% (final chew time), on the first two components of the PCA biplot of attributes and samples.

## 3. Results

### 3.1. Dynamic Product Curves

Sample curves were generated for both sensory tests to understand the effect of hydrocolloids in pureed carrots and to compare the two methods for the dynamic textural properties. These curves are in standardized format where the x-axis no longer represents the actual processing time but is the percentage of oral processing time. The Y-axis represents the dominance rate in TDS and citation proportion in case of TCATA curves. Dominance rate or citation proportion is the sum of all responses divided by the number of times the sample was evaluated, at a particular point of time.

#### 3.1.1. TDS Curves

**Figure 1:**
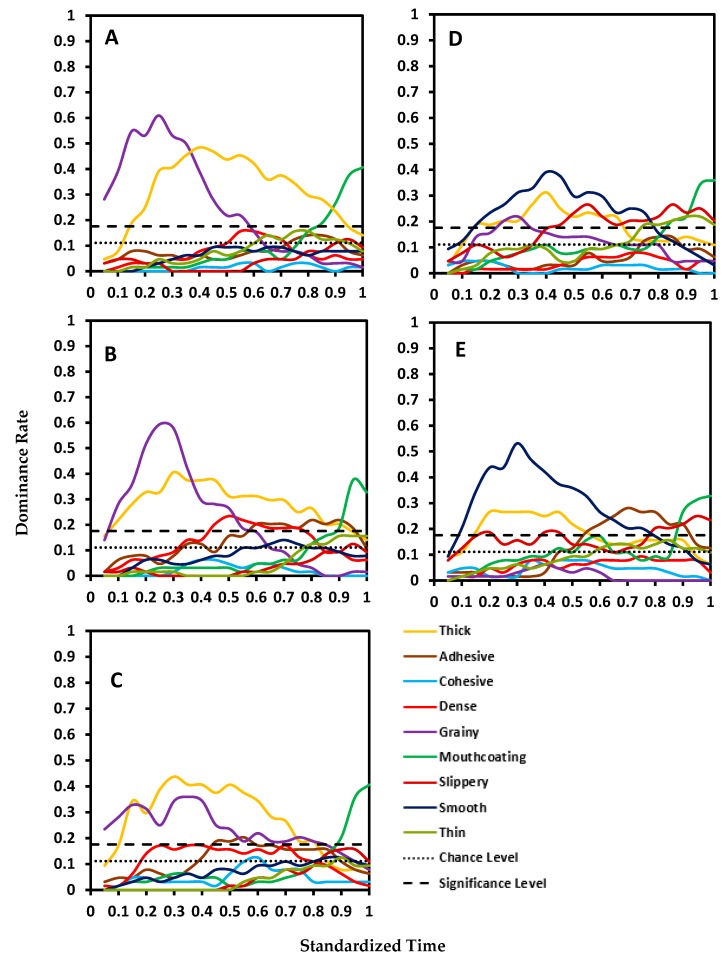
Standardized TDS curves of five pureed carrot matrices made with starch and xanthan: (**A**) control (no starch/xanthan), (**B**) 0.4% starch, (**C**) 0.8% starch, (**D**) 0.2% xanthan, and (**E**) 0.4% xanthan.

**Figure 2:**
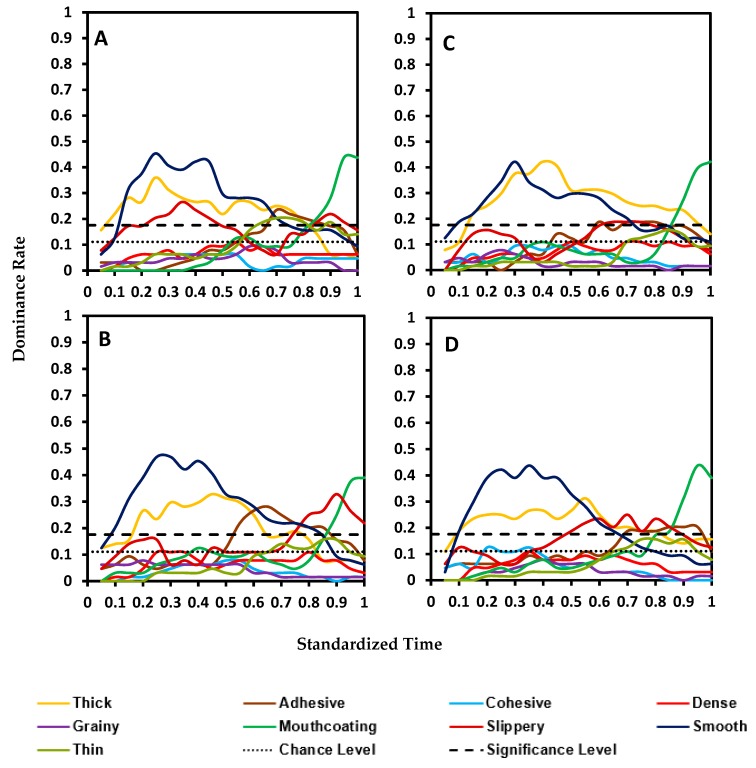
Standardized TDS curves of four pureed carrot matrices made with starch and xanthan blends (S—starch, X—xanthan, number represent the w/w percent concentration in pureed carrots): (**A**)—S0.4/X0.2, (**B**)—S0.4/X0.4, (**C**)—S0.8/X0.2, and (**D**)—S0.8/X0.4.

TDS curves are shown in Figure 1 and Figure 2. The control sample (with no added hydrocolloid) had three dominant attributes during the entire oral processing time—grainy, thick, and mouthcoating (Figure 1A). This was similar for S0.4 (Figure 1B) but there were two more attributes perceived as dominant—dense and adhesive—at very low dominance rates. These were very close to the significance limit of 0.18. For both the samples, grainy had the highest dominance rate for the initial one-third of the OPT at which point thick became dominant until almost the end of the mastication time. Mouthcoating became dominant at approximately 80% of the mastication time. For S0.8 (Figure 1C), the dominant attributes were same as in the control sample with an additional attribute of adhesive being above the significance limit for a small duration during the mid-mastication time (45%–60% OPT).

Pureed carrots matrices made with xanthan showed a different set of attributes as dominant than the control sample. Thick and smooth were the dominant attributes for X0.2 for the majority of the OPT (Figure 1D). Slippery was dominant after the initial one-third chewing time to the end. Similar to the starch samples, mouthcoating was dominant after 80% OPT. Grainy was dominant for a very short time in the first quarter. Similar to the profile of X0.2, pureed carrots made with X0.4 (Figure 1E) was perceived as smooth for most part of chewing time. The other dominant attributes above the significance limit were thick, slippery, adhesive, and mouthcoating. Adhesive was dominant in the third quarter, while slippery and mouthcoating became dominant in the last quarter of OPT and remained that way to the end.

Each of the four blends (Figure 2A–D) had two similar dominant attributes in comparison to the control sample; thick and mouthcoating. The main difference was observed for grainy and smooth. The trend for these was opposite in the blends in comparison to the control. While grainy was dominant and smooth was below the chance limit for the control, in the case of starch–xanthan blends, smooth was a primary dominant attribute and grainy was below the chance limit. The other dominant attributes in all blends were slippery and adhesive.

#### 3.1.2. TCATA Curves

**Figure 3:**
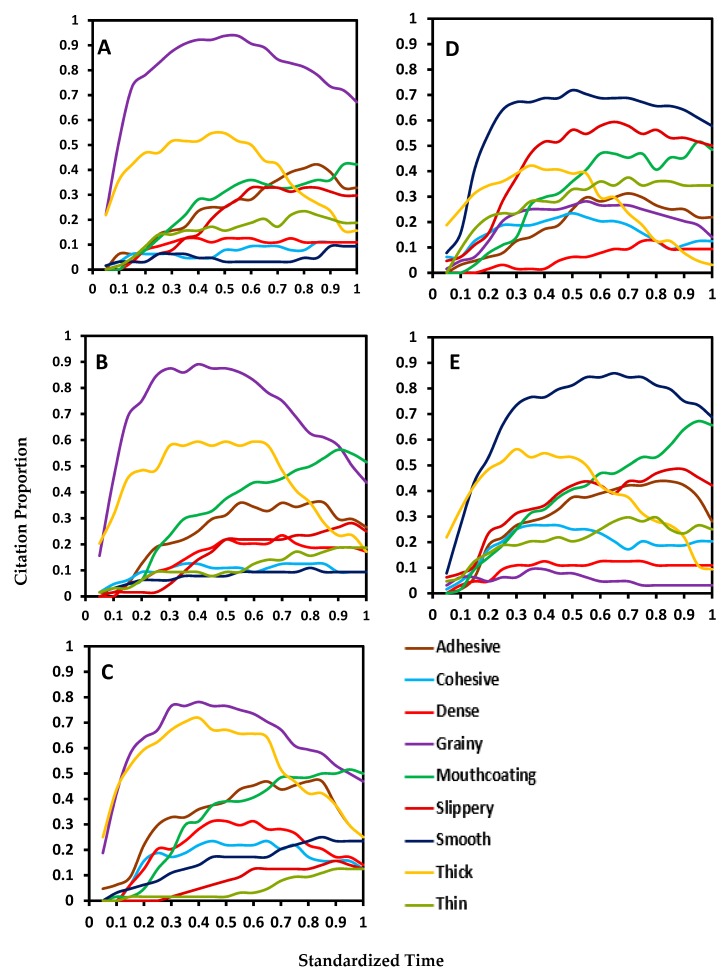
Standardized TCATA curves of five pureed carrot matrices made with starch and xanthan: (**A**)—control (no starch/xanthan), (**B**)—0.4% starch, (**C**)—0.8% starch, (**D**)—0.2% xanthan, and (**E**)—0.4% xanthan.

**Figure 4:**
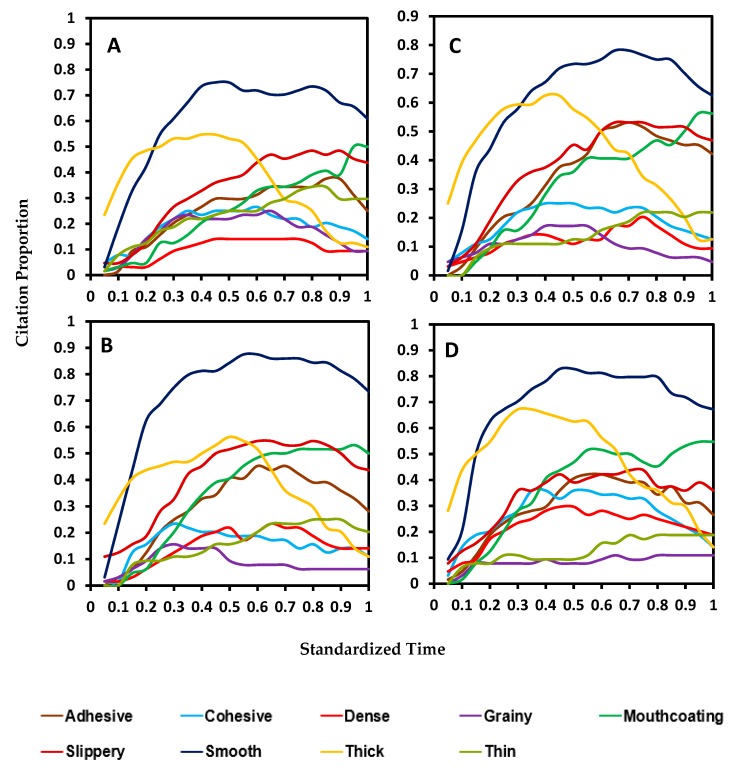
Standardized TCATA curves of four pureed carrot matrices made with starch and xanthan blends (S—starch, X—xanthan, number represent the w/w percent concentration in pureed carrots): (**A**)—S0.4/X0.2, (**B**)—S0.4/X0.4, (**C**)—S0.8/X0.2, and (**D**)—S0.8/X0.4.

The dynamic TCATA curves for the 9 pureed carrot samples are shown in Figure 3 and Figure 4. The control sample had a very high citation proportion for grainy followed by thick (Figure 3A). Grainy was the perceived attribute with highest citation proportions until the end while thick remained high until the third quarter of the OPT. The citation proportion for three attributes—adhesive, mouthcoating, and slippery were higher than thick after 75% of the mastication time. The remaining four attributes of cohesive, dense, thin and smooth had very low citation proportions. Pureed carrots matrices made with S0.4 and S0.8 showed similar trends in dynamic perception as the control sample.

The main difference between both xanthan samples and the control sample was grainy perception. It was very low for X0.2 while it was very high in control sample for most of the mastication time. Xanthan attributed the perception of smooth in the pureed carrot matrix. Slippery and thick were the other two most cited attributes in the first half of the OPT. As the citations decreased for thick, they increased for the attributes of mouthcoating and thin.

Comparison of the blend samples (Figure 4A–D) with the control sample indicated that two attributes, grainy and smooth, had opposite trends in these samples. Grainy was very low for all blends and smooth was high for most of the chewing time. Thick was the only attribute with a similar trend and citation proportion while all the other attributes were perceived at a higher citation than in control.

### 3.2. Difference Curves

**Figure 5:**
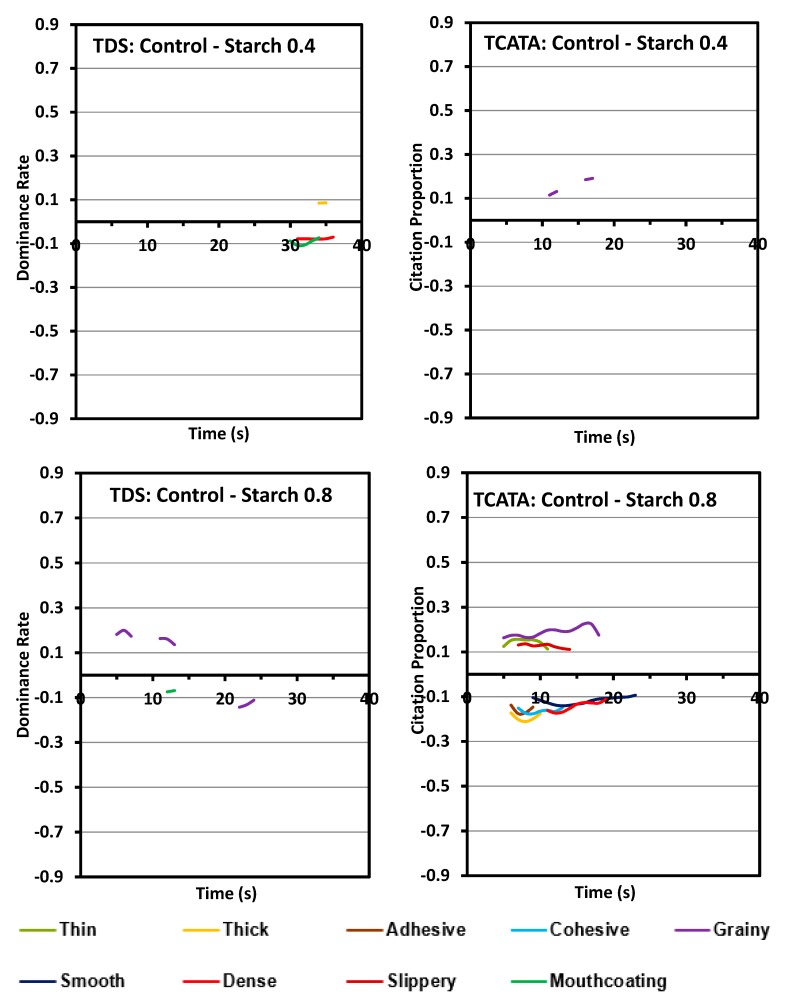
Difference curves of pureed carrot matrices between control and two starch concentrations (0.4% and 0.8% *w*/*w*). TDS (left columns) and TCATA (right columns). Comparisons are made between a pair of samples, the first and second sample are depicted respectively above and below the zero line. Significantly different attributes varying between the samples are shown, calculated at 95% Fischer’s Exact test.

**Figure 6:**
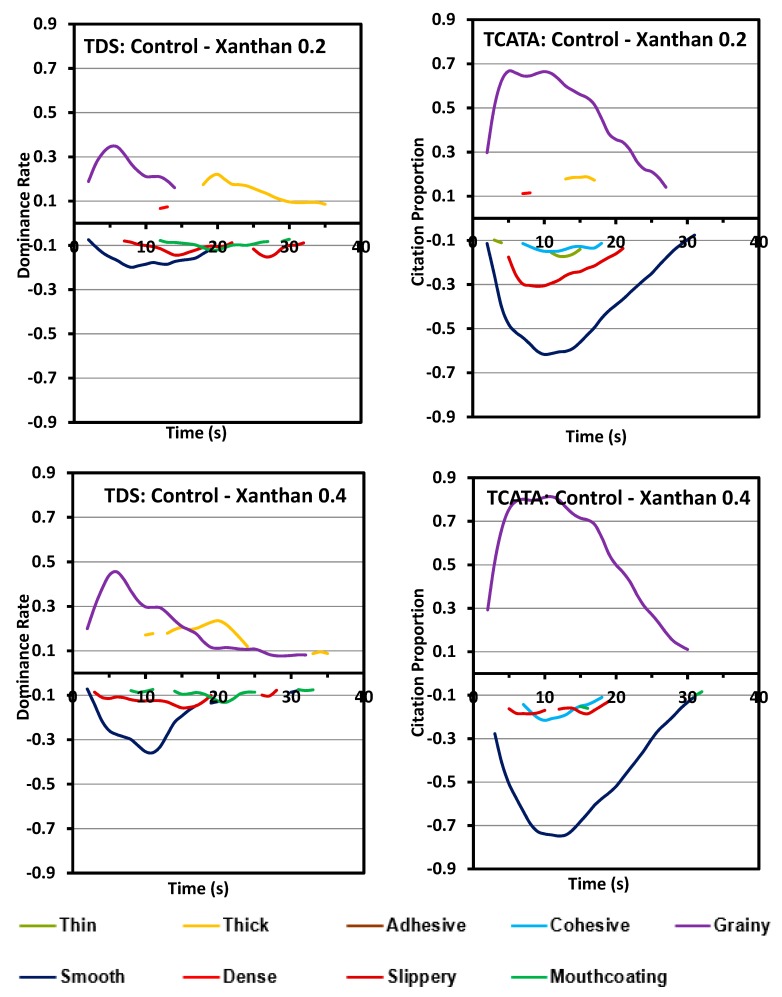
Difference curves of pureed carrot matrices between control and two xanthan concentrations (0.2% and 0.4% *w*/*w*). TDS (left columns) and TCATA (right columns). Comparisons are made between a pair of samples, the first and second sample are depicted respectively above and below the zero line. Significantly different attributes varying between the samples are shown, calculated at 95% Fischer’s Exact test.

The difference curves have been plotted in the unstandardized format with x-axis representing the actual processing times. Difference curves for xanthan and starch products in comparison to the control sample are shown in Figure 5 and Figure 6. As evident from these curves (Figure 5), starch samples (S0.4 and S0.8) had very few differences compared to the control for both TDS and TCATA. The few evident differences are present at a low significance level and for a very low duration. The difference attributes for both the xanthan samples against the control sample are similar with the fact that the difference rate of each different attribute increases with the concentration of xanthan. In general, xanthan samples are more smooth, slippery and have mouthcoating while the control is significantly different for grainy and thick (Figure 6). Some attributes are captured by individual tests such as mouthcoating and thick with TDS, and cohesive with TCATA. The difference curves in TCATA have longer durations for an attribute compared to the TDS because of the nature of the test.

The similarity in texture perception between control and starch containing pureed carrot matrices is also evident from the difference curves between starch and xanthan samples. Most of the significant attributes between starch and xanthan were similar to those between control and xanthan. The differences in texture dynamics due to different concentrations of hydrocolloids used in this study were negligible in both starch and xanthan.

Within the blend samples xanthan was more influential and impacted perceptions more than starch, as shown in the Supplementary Section (Figure S1). Although this is only one example, all blends followed a similar pattern. The differences between blends was also very minimal and comparison between two blends is shown in the Supplementary Section (Figure S2).

### 3.3. Product Map

The product map was obtained by performing a CVA on perception duration of each attribute of the sample by panelist mean score. This biplot gives information regarding product discrimination, where the product means are separated while keeping the panelist scores as close as possible for a specific product mean [36].

**Figure 7:**
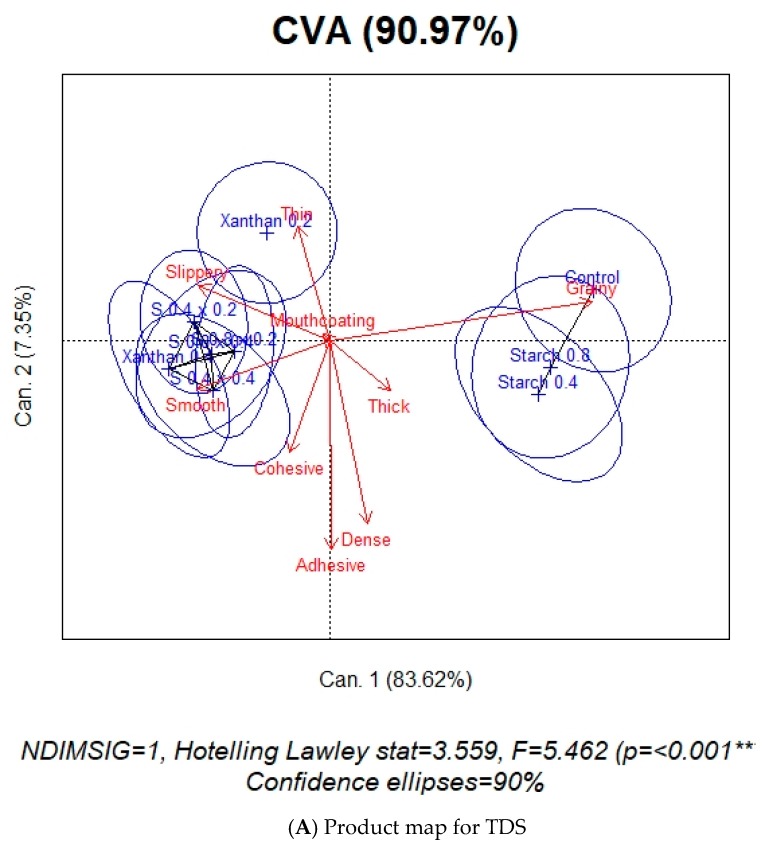

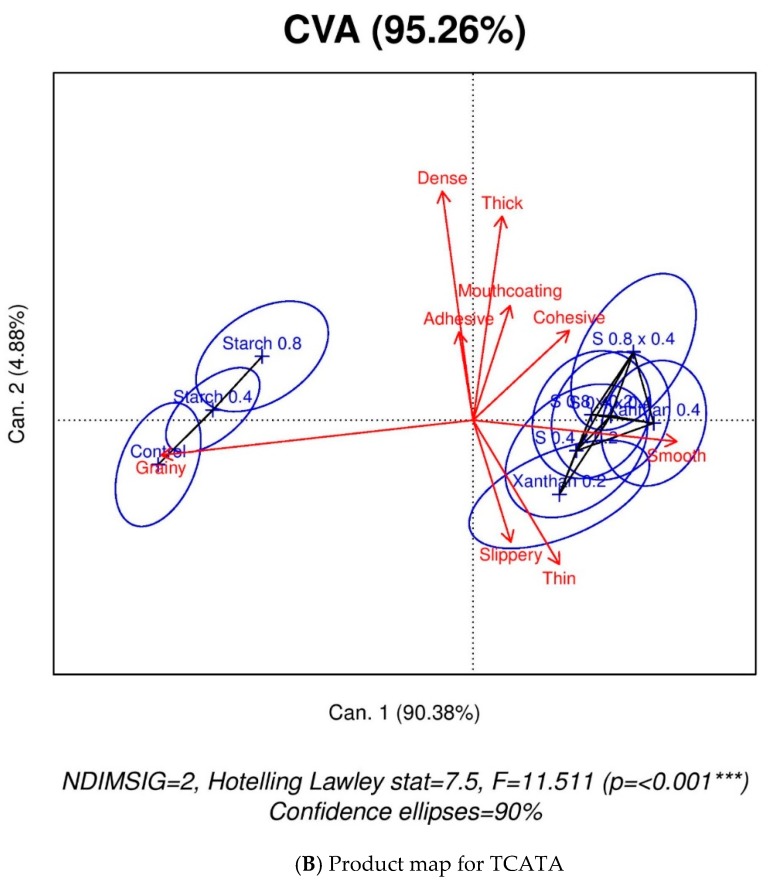
Product map discriminating the nine pureed carrot matrices (*w*/*w*) made with starch, xanthan and their blends, using CVA analysis. (**A**) TDS, (**B**) TCATA. The sample names are in blue, attributes are in red and the connecting black line between the samples is an indicator of their similarities. The samples are control (no starch/xanthan), starch (0.4, 0.8), xanthan (0.2, 0.4), S0.4/X0.2, S0.4/X0.4, S0.8/X0.2, and S0.8/X0.4.

As indicated in Figure 7A, the products were significantly discriminated with *p* < 0.001 and F-statistic of 5.46, for TDS. The first dimension explains a significant amount of variability (84%) of the sample discrimination. The control and both starch samples are characterized by grainy, X0.2 by thin and slippery while most of the others are characterized by smooth, slippery and thin. All blends have textural similarities with X0.4. Since mouthcoating and thick were present in all samples in approximately the same dominance rates, they are either very close to the origin or have a short attribute arrow. In other words, these two do not play a significant role in discriminating the samples.

For the CVA product-attribute biplot of TCATA (Figure 7B), 95% of sample discrimination is accounted by the first two dimensions. The first axis accounting for 90% of the data variability is primarily based on geometrical characteristics of texture—smooth and grainy. The samples are significantly discriminated with *p* < 0.001 and F-statistic of 11.51. As in the case of TDS, control and two starch samples have textural similarities. The remaining samples are closely related in texture perception, being characterized by smooth, slippery, and thin. This is very similar to TDS except for one minor thing. The connecting black line segments in the product maps, between the samples is an indicator of their similarities. According to the TDS output, the four blends are closely related to only X0.4 while, as per the TCATA output, these share similarities with both the xanthan samples. In both tests, the discriminating attributes ae grainy, smooth, slippery, and thin.

### 3.4. Oral Processing Time

The oral processing times were analyzed to see if addition of hydrocolloid and its concentration had an effect on the time to process the purees. No significant differences were found between samples for the oral processing times either by TDS or TCATA. The oral processing times were around 7 s more while performing TDS (range from 30.1–31.7 s) compared to TCATA (22.6–24.2 s). This difference in time between the two techniques could be an artifact of testing. Panelists have to focus on one attribute catching their attention in TDS while in TCATA they have to select all the attributes being perceived, so less focus is needed during the latter sensory evaluation.

### 3.5. Sensory Trajectory

**Figure 8:**
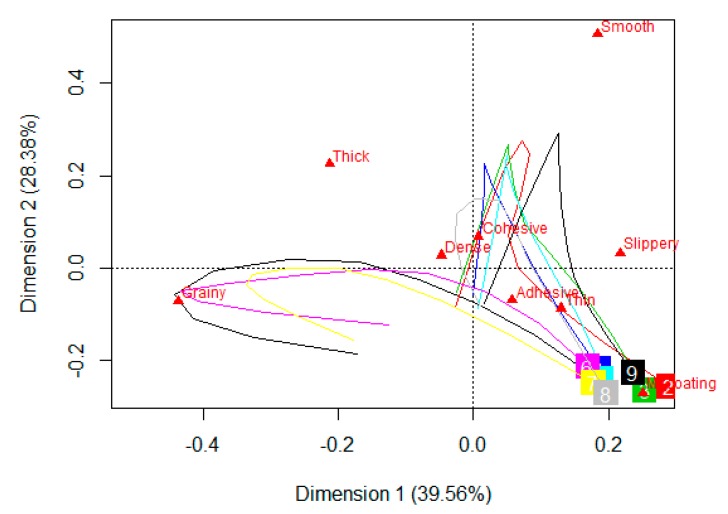
A PCA biplot, depicting the sensory trajectory (TDS) at 11 equally spaced time points during oral processing of nine pureed carrot matrices made with starch (S), xanthan (X), and their blends (S/X). The nine samples are each indicated by a different number and each trajectory ends where the number appears in a colored square box. Some of the numbers are overlapping due to similarities. S—starch, X—xanthan. 1-Control (black), 2-S 0.4/X 0.2 (red), 3-S 0.4/X 0.4 (green), 4-S 0.8/X 0.2 (blue), 5- S 0.8/X 0.4 (turquoise), 6-S 0.4 (pink), 7-S 0.8 (yellow), 8-X 0.2 (grey), and 9-X 0.4 (navy blue).

The sensory trajectories of dominant rates for the nine samples, in TDS, are plotted on the PCA biplot (Figure 8). The first two dimensions explain 68% of the variance. Both the dimensions are guided strongly by geometrical textural parameters, grainy being on the first dimension and smooth on the second dimension. Mouthcoating is the other attribute contributing significantly to the first dimension. All the samples have mouthcoating as the last perceived dominant attribute. The sensory trajectories for the nine samples can be classified into two groups based on their initial perceived attribute and the pathway to reach the end-point of mouthcoating. The first group consisting of control and both starch samples start from grainy perception, moves towards thick, dense, and adhesive, and then ends at mouthcoating. The second group consisting of rest of the six samples follow the path of dense, cohesive, smooth, adhesive, thin, and mouthcoating.

**Figure 9:**
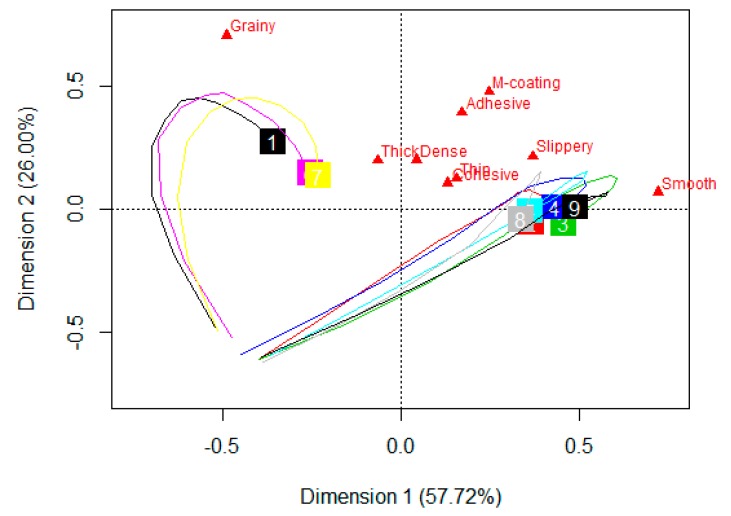
A PCA biplot, depicting the sensory trajectory (TCATA) at 11 equally spaced time points during oral processing of nine pureed carrot matrices made with starch (S), xanthan (X) and their blends (S/X). The nine samples are each indicated by a different number and each trajectory ends where the number appears in a colored square box. Some of the numbers are overlapping due to similarities. S—starch, X—xanthan. 1-Control (black), 2-S0.4/X 0.2 (red), 3-S 0.4/X 0.4 (green), 4-S 0.8/X 0.2 (blue), 5-S 0.8/X 0.4 (turquoise), 6-S 0.4 (pink), 7-S 0.8 (yellow), 8-X 0.2 (grey), and 9-X 0.4 (navy blue).

The sensory trajectories of citation proportions for TCATA data, represented on the first two dimensions of PCA biplot explain 82% of the variance (Figure 9). As observed in TDS, both the dimensions in this case are also strongly influenced by geometrical texture characteristics of smooth (first dimension) and grainy (second dimension). All the other attributes were placed close to each other, specifically thick, dense, thin, and cohesive. The two classification groups of all samples are evident as in the case of TDS with similar samples being in each group. The pathway being followed, showing the progression of attributes from the start to the end is not as clearly marked in the TCATA plot when compared to the TDS plot. All the samples had a common final attribute of mouthcoating in TDS which was not the case in TCATA. This is probably because of the nature of TCATA, which is based on the concept of selecting all the perceived/multiple attributes at a particular time point. Although it could be visualized that the group consisting of control and the two starch samples were mainly in the quadrant for grainy and thick and the other samples had proximity to smooth, thin, cohesive, and slippery perception.

## 4. Discussion

Most published literature investigating the effect of hydrocolloids on sensory properties of semi-solid food systems have looked at starch, fat, and/or protein-based foods such as mashed potatoes, custard, and mayonnaise and have used static methodologies, with a trained panel [39,40]. Our goal was to examine the effect of commonly used hydrocolloids, starch and xanthan, on dynamic textural properties of a carbohydrate-based modified textured food system, as perceived by an untrained panel.

Pureed carrot matrices were made with two concentrations each of starch, xanthan, and four combinations of the starch–xanthan blend. These were compared to the control puree that did not contain any hydrocolloid. The dynamic texture perception of starch added pureed carrots was very similar to the control sample, with three dominant attributes—grainy, thick, and mouthcoating. The duration and the rate of grainy perception decreased with the increase of starch concentration. Addition of xanthan masked the grainy perception and reduced the perception of thick. Xanthan samples were perceived as smooth for most of the processing time followed by slippery, thin, and mouthcoating. Sensory characterization of thickened fluids highlighted similar results where starch had a higher grainy perception while xanthan attributed slipperiness [14]. The smooth perception in pureed carrots attributed by xanthan was similar to that perceived in mashed potatoes where addition of xanthan reduced the perception of granularity and enhanced creaminess [39]. This was attributed to the film being formed by xanthan around the leached amylose. The slippery perception can be associated with increased extensional flow properties of food because of the shear thinning behavior of xanthan, its interaction with salivary mucins and resistance to amylase action [3,7,41]. Probably, the same argument can be given for the xanthan samples being rated low for the perception of thick compared to the control and starch samples.

Synergistic interactions of binary hydrocolloids in the form of blends have been used in various food applications as the desired functional properties can be achieved with a reduced overall concentration of sole hydrocolloid [42,43]. In this research, the four blends of starch and xanthan tested for dynamic texture perceptions were very similar to each other and with the two xanthan samples. Even at low concentrations, xanthan masks the grainy perception of starch as observed in the blend where xanthan was added at one-fourth of starch concentration (S0.8/X0.2).

Addition of starch and xanthan increased the cohesive perception of pureed carrots, fulfilling an important criterion of adding these to the compensatory diets of people with swallowing disorders. Starch and xanthan had no effect on mouthcoating, which was similar in all samples occurring towards the end of the OPT and with almost similar dominance/citation rates. Another highlight of this study is the fact that even when the overall concentration of starch and xanthan in pureed carrots is the highest (S0.8/X0.4), dense is not a dominant attribute.

Since modified texture foods are used as a dietary intervention for people with dysphagia, an important attribute that needs to be examined is ease of swallowing. Cohesiveness and ease of swallowing are interrelated; a cohesive sample is easier to manipulate and swallow [3]. Given the nature of dynamic testing, it was not possible to include ease of swallow as an attribute, however, it should be included in future studies of food oral processing of pureed products.

TDS and TCATA product and difference curves showed similarities in attribute appearance in all samples. The major difference between the two tests were the increased duration and citation rate of attributes in TCATA compared to TDS. This is a function of test methodology. These tests also differentiated the samples in the same way with similar differentiating attributes across the nine samples, as per the CVA results (Figure 7). This is contrary to the findings of other published literature comparing TDS and TCATA. The results of a dynamic sensory study conducted on a range of products indicated better sensory discrimination with TCATA than TDS [31]. Others have concluded that, although the results were similar across TDS, TCATA, and Progressive Profiling, TCATA was felt to provide a complete product description [30]. Similarly, it was concluded that product discrimination was found to be better with TCATA and M-TDS (TDS by one modality such as texture or flavor) than TDS, when studying yogurts varying in flavor and texture [29].

An advantage of using dynamic sensory tests over static tests is the ability to visualize the sensory trajectory which provides information about the succession of perceived attributes over the oral processing time. Others have also shown this in semi-solid foods, gels, and cheese [44,45]. In the current study, the sensory trajectory generated for TCATA was less obvious than the trajectory generated from the TDS data, although the sample groupings was similar in both tests. A clear attribute path was drawn, in TDS, for the movement of a sample starting from the initial attribute to the time it was swallowed as only one attribute was selected at a point of evaluation, unlike TCATA, where multiple attributes can be selected at a particular point of time.

While this study has shown the differences in dynamic perceptions of starch and xanthan-based products, one limitation is that the attribute list was not randomized across the panelists. As suggested by Pineau et al [21], the attribute order can create a small bias on the time of selection where attributes at the top are selected before the ones at the bottom of the list. However, in the current study, attributes were listed in a 3x3 matrix with thick, thin, and adhesive at the top of the matrix. Attributes that were most often considered dominant/perceived by participants were smooth, mouthcoating, and grainy, indicating that a bias toward the attributes at the top of the list was not evident. Another limitation is that the subjects performing the tests were not the population for which modified textured foods exist. Participants used in this study were selected from a healthy population of university students and while this may have biased the results, collecting dynamic perceptions using individuals with dysphagia may not be possible to due cognitive difficulties and other health concerns. To date, no one has studied dynamic sensory perceptions with this population.

## 5. Conclusions

The purpose of this research was first, to examine the effect of hydrocolloid addition on sensory properties of pureed carrots. The concentration of hydrocolloid added to the carrots was less influential on dynamic sensory properties than the type of hydrocolloid used. Corn starch addition contributed a grainy texture while xanthan contributed a smoothness to the product. When blending the two hydrocolloids, xanthan was more influential than corn starch on impacting sensory perception. The second objective was to compare results between two dynamic tests. Based on the results observed in this research, both TDS and TCATA capture similar information indicating that either of the two tests can be used to examine the dynamic nature of sensory perception of a modified texture food such as pureed foods.

## Figures and Tables

**Table 1:** Concentrations of starch, xanthan, and starch/xanthan blends used while making pureed carrot matrices. (S—Starch, X—Xanthan, S/X—Blend of Starch and Xanthan).

Treatments	Sample	Starch (*w*/*w* %)	Xanthan (*w*/*w* %)
1	Control	0	0
2	S0.4	0.4	0
3	S0.8	0.8	0
4	X0.2	0	0.2
5	X0.4	0	0.4
6	S0.4/X0.2	0.4	0.2
7	S0.8/X0.2	0.8	0.2
8	S0.4/X0.4	0.4	0.4
9	S0.8/X0.4	0.8	0.4

**Table 2:** Attribute definition with reference foods used for Temporal Dominance of Sensations (TDS) and Temporal Check-All-That-Apply (TCATA) test.

Attribute	Definition	Reference Food
Adhesive	The degree of sample that sticks to oral surfaces (like palate, tongue, teeth).	WOWBUTTER Creamy, Canada
Cohesive	Tendency of the product mass to stay together in one piece.	Gerber^®^ Rice & Banana Baby Cereal (Add Water), Nestle, Canada
Dense	The compactness of the food product, or how solid it feels when you are orally processing it.	PHILADELPHIA Original Cream Cheese, Kraft Canada Inc.
Grainy	The presence of small, rough particles in the mouth surface.	Unsweetened Applesauce, Mott’s^®^ USA
Thick	This is related to the viscosity of the sample, how hard/easy (amount of effort needed) it is to move the sample in the mouth while orally processing it.	Nordica Smooth Plain Cottage Cheese, Gay Lea^®^, Canada
Thin	Product readily flows, related to viscosity of the sample (the opposite of thick).	0% Vanilla Yogurt (Mixed with water 1:1), IÖGO, Canada
Mouthcoating	The amount of film on the mouth surfaces, the coating on the oral surfaces inside the mouth.	Homestyle Mashed Potato, Betty Crocker, USA. Made as per instructions but used 35% cream and 2 Tbsp Margarine instead of milk.
Slippery	How the food slides/slips down to the back of the mouth.	Miracle Whip Regular, Kraft Canada Inc.
Smooth	Velvety feeling of the sample in your mouth. It is the absence of surface particles (the opposite of grainy).	Greek Yogurt (Vanilla Bean 5%), LIBERTÉ, Canada

## Supplementary Materials

The following are available online at https://www.mdpi.com/2304-8158/8/6/184/s1, Table S1: IDDSI guidelines for modified textured foods, Figure S1: Difference curves TDS (left columns) and TCATA (right columns). Comparisons are made between a pair of samples, the first and second sample are depicted respectively above and below the zero line. Significantly different attributes varying between the samples are shown, calculated at 95% Fischer’s Exact test, Figure S2: Difference curves TDS (left columns) and TCATA (right columns). Comparisons are made between a pair of samples, the first and second sample are depicted respectively above and below the zero line. Significantly different attributes varying between the samples are shown, calculated at 95% Fischer’s Exact test.
