# Supplementary material for: Characterizing the Dynamic Textural Properties of Hydrocolloids in Pureed Foods—A Comparison Between TDS and TCATA
Source: Foods. 2019 May 30;8(6):184. doi: 10.3390/foods8060184 (PMC6617281; doi:10.3390/foods8060184)
Supplement: Supplementary file 1 [file foods-08-00184-s001.pdf]

Table S1. International Dysphagia Diet Standardization Initiative (IDDSI) guidelines for modified textured foods

Test Name	Description
Fork Drip Test	Food sits in a mound above fork, a tail forms, but food does not continuously flow through fork tines
Fork Pressure Test	Visible pattern on surface of food, food retains visible indentation, has no lumps
Spoon Tilt	Food holds shape on spoon, slides off spoon with little left, spreads slightly and / or slumps slowly on flat plate
Finger Test	Food slides between fingers smoothly and easily, noticeable residue remains

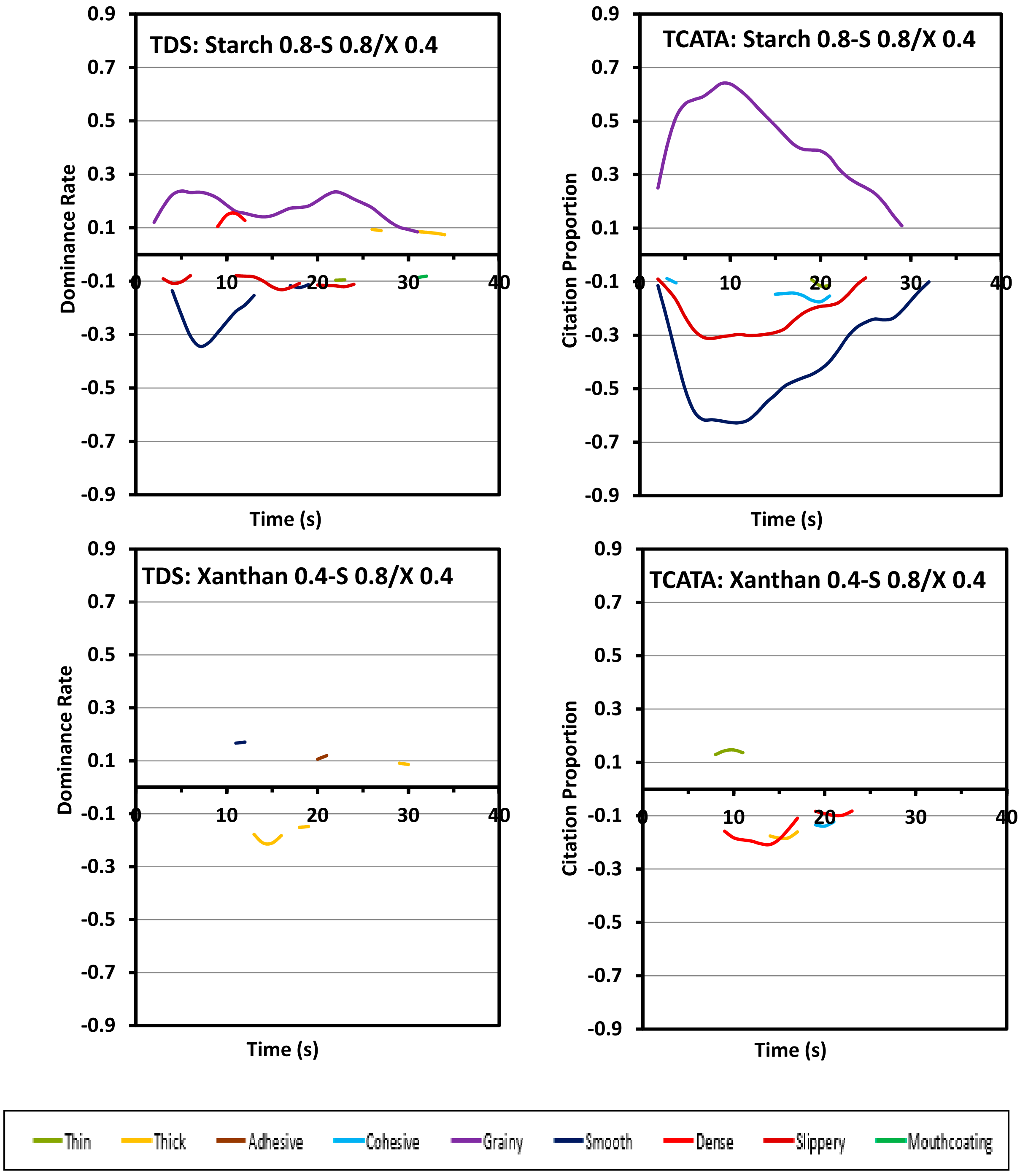

Figure S1. Difference curves for pureed carrot matrices made with either starch (0.8% w/w) or xanthan (0.4% w/w) and compared to their blend (S 0.8/X 0.4). TDS (left columns) and TCATA (right columns). Comparisons are made between a pair of samples, the first and second sample are depicted respectively above and below the zero line. Significantly different attributes varying between the samples are shown, calculated at 95% Fischer's Exact test.

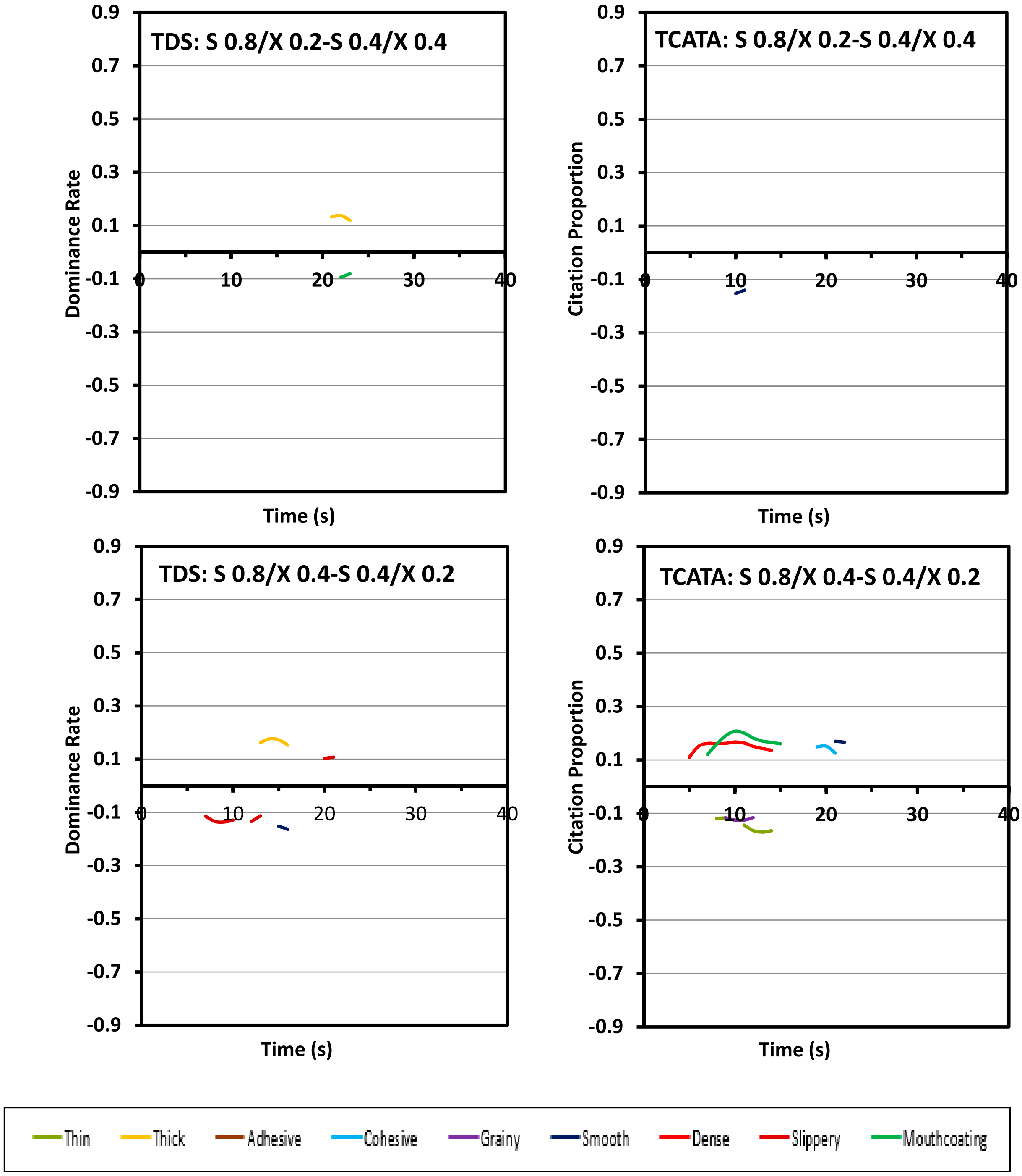

Figure S2. Difference curves of two pureed carrot matrices made with different blends of starch and xanthan. TDS (left columns) and TCATA (right columns). Comparisons are made between a pair of samples, the first and second sample are depicted respectively above and below the zero line. Significantly different attributes varying between the samples are shown, calculated at 95% Fischer's Exact test.
